# Supplementary material for: An updated sulfate transporter phylogeny uncovers a perennial-specific subgroup associated with lignification
Source: Tree Physiol. 2025 Jul 11;45(13):114–28. doi: 10.1093/treephys/tpaf080 (PMC12666378; doi:10.1093/treephys/tpaf080)
Supplement: SUPPLEMENTARY_DATA_tpaf080 [file supplementary_data_tpaf080.docx]

**SUPPLEMENTARY DATA**

Table S1. List of SULTR sequences used in the phylogenetic analysis

Table S2. List of RNA-Seq datasets from *Populus tremula* × *alba* stem tissues used in coexpression analysis

Table S3. Genes and their Pearson correlation coefficients with *PtaSULTR3.2a* and *PtaSULTR3.4a*

Table S4. List of RNA-Seq datasets from transgenic *Populus trichocarpa*
